# Whole Genome Sequencing Reveals Biopesticidal Origin of *Bacillus thuringiensis* in Foods

**DOI:** 10.3389/fmicb.2021.775669

**Published:** 2022-01-12

**Authors:** Michael Biggel, Danai Etter, Sabrina Corti, Peter Brodmann, Roger Stephan, Monika Ehling-Schulz, Sophia Johler

**Affiliations:** ^1^Vetsuisse Faculty, Institute for Food Safety and Hygiene, University of Zurich, Zurich, Switzerland; ^2^Kantonales Labor Basel-Stadt, Basel, Switzerland; ^3^Department of Pathobiology, Functional Microbiology, Institute of Microbiology, University of Veterinary Medicine Vienna, Vienna, Austria

**Keywords:** *Bacillus thuringiensis*, *B. cereus* group, foodborne outbreaks, biopesticide, enterotoxin

## Abstract

*Bacillus thuringiensis* is a microbial insecticide widely used to control agricultural pests. Although generally regarded as safe, *B. thuringiensis* is phylogenetically intermingled with the foodborne pathogen *B. cereus sensu stricto* and has been linked to foodborne outbreaks. Limited data on the pathogenicity potential of *B. thuringiensis* and the occurrence of biopesticide residues in food compromise a robust consumer risk assessment. In this study, we analyzed whole-genome sequences of 33 *B. thuringiensis* isolates from biopesticides, food, and human fecal samples linked to outbreaks. All food and outbreak-associated isolates genomically matched (≤ 6 wgSNPs; ≤ 2 cgSNPs) with one of six biopesticide strains, suggesting biopesticide products as their source. Long-read sequencing revealed a more diverse virulence gene profile than previously assumed, including a transposase-mediated disruption of the promoter region of the non-hemolytic enterotoxin gene *nhe* and a bacteriophage-mediated disruption of the sphingomyelinase gene *sph* in some biopesticide strains. Furthermore, we provide high-quality genome assemblies of seven widely used *B. thuringiensis* biopesticide strains, which will facilitate improved microbial source tracking and risk assessment of *B. thuringiensis*-based biopesticides in the future.

## Introduction

*Bacillus thuringiensis* is one of the most popular plant protection agents worldwide, accounting for 90% of the bioinsecticide market (Chattopadhyay et al., 2004). The organism synthesizes parasporal crystals as well as other entomopathogenic factors with insecticidal activity, leading to its widespread application in agriculture and vector control (Chattopadhyay et al., 2004; Jouzani et al., 2017). *B. thuringiensis*-based biopesticides represent an important alternative to broad-spectrum chemical pesticides, as they exhibit a high degree of host specificity, with only limited harmful effects to non-target species (Lacey et al., 2015). In the coming decade, the use of these already highly popular biopesticides will likely increase further. The agrosystems transformation and major political agendas are committed to promoting healthy foods and preserving biodiversity. Major drivers are the United Nations’ Sustainable Development Goals and the Farm to Fork Strategy, which is a vital part of the European Green Deal: By the year 2030, a decrease of chemical pesticides by 50% is envisaged, while at the same time increasing organic farming to 25% of total farmland. These ambitious agendas will inevitably generate additional demand for biopesticides and may substantially boost the use of *B. thuringiensis*-based formulations.

However, *B. thuringiensis* lacks Qualified Presumption of Safety (QPS) status and the role of *B. thuringiensis* as a causative agent of diarrheal disease is controversially discussed (McIntyre et al., 2008; EFSA BIOHAZ Panel, 2016; Raymond and Federici, 2017; Bonis et al., 2021). *B. thuringiensis* belongs to the *B. cereus sensu lato* group and is genetically intermingled with the widely recognized foodborne pathogen *B. cereus sensu stricto* (Ehling-Schulz et al., 2019; Carroll et al., 2020b). In addition, *B. thuringiensis* strains from biopesticides can express medium to high levels of enterotoxins and were linked to outbreaks of diarrheal disease (Johler et al., 2018; Schwenk et al., 2020; Bonis et al., 2021).

*B. thuringiensis* is ubiquitous in the environment, which may result in the contamination of agricultural products (Ankolekar et al., 2009; Kim et al., 2017; Bağcıoğlu et al., 2019). Thus, it has been suggested that *B. cereus*-like spores detected on foods largely stem from contamination with naturally occurring wild-type strains (Raymond and Federici, 2017). While there are indications that residues from biopesticides could also be detected (Frederiksen et al., 2006; Hendriksen and Hansen, 2006; Zhou et al., 2008; Johler et al., 2018; Frentzel et al., 2020; Bonis et al., 2021), the extent to which these are present in foods is unclear. In this study, we aimed to utilize genomic analysis to assess a potential link of biopesticides to *B. thuringiensis* strains detected on foods.

## Materials and Methods

### Isolation of *B. cereus sensu lato* From Food Samples

A total of 100 samples of tomatoes, bell peppers, and salads from a central retail distribution facility in Switzerland were screened for *B. cereus sensu lato* (Table 1 and Supplementary Table 1). 10 g of each sample was added to 90 g peptone salt solution (0.1% enzymatic casein digest) and homogenized in a stomacher@ 400 circulator (Seward) for 60 s. Subsequently, *B. cereus s.l.* counts were determined by plating in serial dilutions on selective media (Mannitol-Egg Yolk-Polymyxin [MYP] agar) and incubated at 30°C overnight. Up to five presumptive *B. cereus s.l.* colonies (mannitol negative, phospholipase C positive phenotype) per sample were selected and whenever different colony morphologies were observed, these were included in the selection. Isolates were purified and preserved in glycerol stocks at −80°C until further characterization.

**TABLE 1:** Prevalence of *B. thuringiensis* and other *B. cereus* group members in 100 food samples collected at the retail level in Switzerland.

Product	N samples	Samples positive for any *B. cereus s. l.*	Total number of *B. cereus s. l.* isolates screened for a crystal-producing phenotype	Bacterial loads in positive samples (CFU/g)	*B. thuringiensis* *^a^*		Other *B. cereus s. l.* group members	
					Positive samples (%)	Total number of isolates (% of all investigated isolates)	Positive samples (%)	Total number of isolates (% of all investigated isolates)
Tomato	39	7 (18%)	26	100—34,000 (median 500)	7 (18%)	26 (100%)	0 (0%)	0 (0%)
Bell pepper	21	4 (19%)	12	100—3,600 (median 550)	4 (19%)	12 (100%)	0 (0%)	0 (0%)
Endives	18	10 (56%)	26	100—1,800 (median 300)	1 (6%)	3 (12%)	9 (50%)	23 (88%)
Rocket salad	11	2 (18%)	8	25,000 and 40,000	2 (18%)	8 (100%)	0 (0%)	0 (0%)
Baby spinach	7	2 (29%)	4	100 and 1,300	0 (0%)	0 (0%)	2 (29%)	4 (100%)
Baby leaf salad	4	2 (50%)	2	100 each	0 (0%)	0 (0%)	2 (50%)	2 (100%)
Total	100	27 (27%)	78	100—40,000 (median 400)	14 (14%)	49 (63%)	13 (13%)	29 (37%)

*^a^B. thuringiensis was identified by phase-contrast microscopy based on a crystal-producing phenotype.*

### Screening for Parasporal Crystals Characteristic for *Bacillus thuringiensis*

Pure cultures of all isolates were grown on T3 agar (Travers et al., 1987) for 3 days at 30°C. A tiny amount of colony material was resuspended in 10 μL sterile water and screened for crystals of diamond, spherical or bipyramidal shape using phase-contrast microscopy (EFSA BIOHAZ Panel, 2016). Isolates exhibiting characteristic parasporal crystals were defined as *B. thuringiensis*.

### Metabolomic Fingerprinting by Fourier-Transform Infrared Spectroscopic Analysis

All *B. thuringiensis* were screened by FT-IR spectroscopy to detect clusters of related isolates and select a subset of strains for whole-genome sequencing. To this end, metabolic fingerprints of all isolates were generated as previously described (Ehling-Schulz et al., 2005). Briefly, isolates were cultivated as lawns on tryptone soy agar (TSA) (Oxoid) at 25°C for 24 h. Subsequently, one loop of cell material was suspended in 300 μL sterile deionized water, subjected to ultrasonication for 5 × 1 s at 100% power with a Bandelin Sonopuls HD2200 (Bandelin electronic), and the bacterial suspension was spotted on a zinc selenite (ZnSe) optical plate. After drying the plates, infrared absorption spectra were recorded using an HTS-XT microplate adapter coupled to a Tensor 27 FT-IR spectrometer (Bruker Optics). Spectra in the range of 4,000–500 cm^–1^ were acquired in transmission mode with the following parameters: 6 cm^–1^ spectral resolution, zero-filling factor 4, Blackmann-Harris 3-term apodization, and 32 interferograms were averaged with background subtraction for each spectrum. The quality of FTIR spectral data was evaluated using the OPUS software (version 7.5; Bruker Optics). Second derivatives were calculated using the Savitzky-Golay algorithm with 11 smoothing points and the derivative spectra were unit vector normalized for further data processing. The so-called fingerprint region (spectral region of 1,500–800 cm^–1^) was chosen for chemometric analyses. Hierarchical cluster analysis (HCA) was carried out using Ward’s algorithm.

### Whole-Genome Sequencing and Genome Analyses

For short-read sequencing, genomic DNA was extracted using the DNeasy Blood & Tissue Kit (Qiagen). Libraries were prepared using the Nextera DNA Flex Library Preparation Kit (Illumina), and sequencing was performed on the Illumina MiniSeq platform with 2 × 150 bp paired-end chemistries. Illumina read adapters and low-quality bases were trimmed with TrimGalore v0.6.6^1^ and quality metrics computed using FastQC v0.11.9^2^. Draft genomes were assembled using SPAdes v3.14.1 (Bankevich et al., 2012) implemented in shovill v1.1.0^3^. Additionally, all seven biopesticide isolates obtained during this study were long-read sequenced. For nanopore sequencing, genomic DNA was extracted using the MasterPure Complete DNA and RNA Purification Kit (Lucigen). Multiplex libraries were prepared using the SQK-LSK109 ligation sequencing kit with the EXP-NBD114 native barcoding expansion kit (Oxford Nanopore Technologies). Libraries were sequenced on a MinION Mk1B device using the FLO-MIN106 (R9) flow cell (Oxford Nanopore Technologies). Adapters were trimmed using Porechop v0.2.4^4^ and quality assessed with LongQC v1.2.0 (Fukasawa et al., 2020). Hybrid assemblies were produced with Unicycler 0.4.8 (Wick et al., 2017). Assembly quality metrics were assessed using QUAST v5.0.3 (Gurevich et al., 2013).

Multi-locus sequence types (MLST), *panC* types, and the presence of virulence genes were determined using BTyper3 and srst2 0.2.0 (Inouye et al., 2014; Carroll et al., 2020a). The completeness of virulence genes and their promoter regions was additionally investigated using BLAST (Altschul et al., 1990) implemented in abricate 1.0.1^5^ and putative disruptions manually inspected in assemblies annotated with prokka v1.14.6 (Seemann, 2014). Prophages were detected using PHASTER (Arndt et al., 2016). *sph* genes disrupted by the insertion of a BceA1-like bacteriophage were identified by screening genomes for the sequence of open reading frame BG08_RS31255 (strain HD-1, GCF_000835235.1;>99% alignment coverage,>99% sequence identity in BLAST), which contains elements of *sph* and of a site-specific integrase from the bacteriophage.

### SNP Analysis and Phylogenies

Core genome SNPs (cgSNPs) among CC8 and CC23 isolates were detected from Illumina read data using the CFSAN SNP pipeline v2.2.1 (Davis et al., 2015) with chromosomes of strains HD-1 (CC8, GCF_000835235.1), and BGSC 4Q7rifR (CC23, GCF_013113775.1) as references, respectively. Phages, IS elements, and repeat regions in reference chromosomes were identified using phastaf v0.1.0^6^, ISEScan v1.7.2.3 (Xie and Tang, 2017), and NUCmer v3.1 (Marçais et al., 2018) and masked prior to read-mapping. cgSNP, i.e., SNP sites present in all genomes, were extracted from the SNP alignment by removing missing sites using SNP-sites v2.5.1 (Page et al., 2016), and core-genome SNP (cgSNP) distances were determined with snp-dists v0.7.0^7^. Maximum-likelihood phylogenetic trees were constructed from the SNP alignment using IQ-TREE v2.0.3 (Nguyen et al., 2015) with the generalized time-reversible (GTR) model and gamma distribution with 100 bootstraps. The number of invariant sites was estimated from core genome alignments generated with parsnp v1.5.3 (Treangen et al., 2014) and passed to IQ-TREE. A phylogenetic tree of all 33 isolates was created using IQ-TREE directly from a core genome alignment generated with parsnp.

Whole-genome SNPs (wgSNPs) were detected by mapping reads of food/human isolates to the assembly of the phylogenetically closest biopesticide isolate (without any masking) using the CFSAN SNP pipeline. Read data of the respective reference biopesticide isolate were included in the pipeline as a reference to calculate wgSNP distances. The percentage of reference assembly bases covered by sequencing reads was determined with BBMap^8^.

## Results

### Occurrence of *Bacillus thuringiensis* in Foods at the Retail Level in Switzerland

A total of 100 food samples (tomatoes, bell pepper, endives, rocket salad, baby spinach, and baby leaf salad) were collected at the retail level. Presumptive *B. cereus s. l.* isolates were identified in 27 samples. From these 27 samples, a total of 78 isolates (up to 5 isolates per sample) were screened by phase-contrast microscopy. A crystal-producing phenotype, characteristic for *B. thuringiensis*, was identified for 49 isolates (63%) from 14 food samples (14%). Positive samples included tomatoes (7/39, 18%), bell pepper (4/21, 19%), rocket salad (2/11, 18%), and endives (1/18, 6%) (Table 1). No *B. thuringiensis* was detected in samples from baby spinach (*n* = 7) and baby leaf salad (*n* = 4). Remarkably, all investigated *B. cereus s. l.* isolates from tomatoes (*n* = 26), bell pepper (*n* = 12), and rocket salad (*n* = 8) were identified as *B. thuringiensis*. In the 14 samples positive for *B. thuringiensis*, presumptive *B. cereus* counts ranged from 100 to 40,000 CFU/g and were thus below the alert threshold of 10^5^ CFU/g (Table 1 and Supplementary Table 1). In 13 food samples, only non-crystal-producing *B. cereus s. l.* isolates were detected. These were particularly common in endives (9/18, 50%) and baby leaf salad (2/4, 50%) and reached bacterial loads of up to 1,800 CFU/g.

### Genetic Similarity of *Bacillus thuringiensis* Isolates From Biopesticide Products, Food, and Outbreaks

*B. cereus s. l.* isolates from all food samples were subjected to FT-IR spectroscopy for dereplication and selection of representative isolates to be included in the WGS analysis. *B. thuringiensis* isolates from the biopesticide products Agree, B401, Delfin, DiPel, Novodor, Solbac, and XenTari were included as references. Closely related isolates were grouped into clusters using Ward’s hierarchical clustering method. Out of each cluster containing *B. thuringiensis*, either all (for small clusters and singletons) or a minimum of three isolates from distinct samples were subjected to WGS analysis for further characterization. Two samples were represented by two isolates each which fell into different FT-IR clusters (Supplementary Table 2).

Overall, whole-genome sequences of 13 biopesticide product isolates, 15 food isolates, and 5 isolates linked to two foodborne outbreaks were investigated to identify potential matches of food/outbreak isolates with biopesticide strains. Of these 33 *B. thuringiensis* isolates, 27 were sequenced as part of this study (Table 2). These included 9 food isolates obtained from 7 samples as part of this study. To obtain a more comprehensive picture of possible links between biopesticide strains, food isolates, and outbreaks, the collection was supplemented with 18 previously described isolates originating from biopesticide products (*n* = 7), food (*n* = 9), or human fecal samples (*n* = 2) (Johler et al., 2018). Three of these food isolates and the two fecal isolates were linked to foodborne outbreaks of diarrheal disease (Johler et al., 2018); three food isolates were recovered during controls by a cantonal laboratory (CH_69, CH_72, CH_81) from lasagna, vegetable juice, and sauce; two isolates were obtained from honey during the production process; and one isolate was obtained from tarragon at the retail level. Furthermore, publicly available WGS data of 6 previously sequenced isolates from additional biopesticide products (Bonis et al., 2021) were included for genomic analyses.

**TABLE 2:** Bacterial isolates selected for whole-genome sequencing (WGS) and genome assembly metrics.

Strain ID	Source of isolation	Assembly level	Assembly N50	Average read depth (Illumina data)	Genome accession number	References isolation	References WGS
CH_181	Biopesticide (XenTari; strain ABTS-1857)	Complete	5,656,085	52x	GCA_020809245.1	Johler et al., 2018	This study
CH_186	Biopesticide (Agree; strain GC-91)	Near-complete	5,705,790	61x	GCA_020809205.1	Johler et al., 2018	This study
P05_2	Biopesticide (B401/Certan; strain ABTS-1857/B401)	Near-complete	5,713,893	81x	GCA_020809125.1	Johler et al., 2018	This study
CH_133	Biopesticide (Solbac; strain BMP144)	Near-complete	5,496,624	33x	GCA_020809185.1	Johler et al., 2018	This study
CH_187	Biopesticide (Novodor; strain NB-176)	Near-complete	5,427,105	49x	GCA_020809165.1	Johler et al., 2018	This study
CH_164	Biopesticide (Delfin; strain SA11)	Near-complete	5,676,138	68x	GCA_020809145.1	Johler et al., 2018	This study
CH_183	Biopesticide (DiPel; strain ABTS-351)	Near-complete	5,674,619	91x	GCA_020809105.1	Johler et al., 2018	This study
18SBCL484	Biopesticide (Vectobac; strain AM65-52)	Draft	148,443	96x	GCA_020775355.1	Bonis et al., 2021	Bonis et al., 2021
18SBCL483	Biopesticide (Aquabac; strain BMP144)	Draft	102,272	101x	GCA_020775305.1	Bonis et al., 2021	Bonis et al., 2021
18SBCL614	Biopesticide (Costar; strain SA-12)	Draft	61,087	101x	GCA_020775315.1	Bonis et al., 2021	Bonis et al., 2021
18SBCL216	Biopesticide (Belthirul; strain PB-54)	Draft	68,139	93x	GCA_020775275.1	Bonis et al., 2021	Bonis et al., 2021
18SBCL421	Biopesticide (Lepinox; strain EG2348)	Draft	58,711	100x	GCA_020775295.1	Bonis et al., 2021	Bonis et al., 2021
18SBCL218	Biopesticide (Delfin Jardin; strain SA-11)	Draft	57,618	97x	GCA_020775255.1	Bonis et al., 2021	Bonis et al., 2021
AGES_2_27_S	Human faces (linked to foodborne outbreak, Lower Austria)	Draft	70,892	85x	GCA_020775395.1	Johler et al., 2018	This study
AGES_6_27_S	Human faces (linked to foodborne outbreak, Lower Austria)	Draft	72,601	88x	GCA_020775365.1	Johler et al., 2018	This study
CH_65	Food (tarragon)	Draft	59,871	52x	GCA_020775435.1	Johler et al., 2018	This study
CH_69	Food (precooked lasagna)	Draft	79,950	68x	GCA_020775725.1	Johler et al., 2018	This study
CH_72	Food (vegetable juice)	Draft	74,180	58x	GCA_020775425.1	Johler et al., 2018	This study
CH_81	Food (precooked sauce)	Draft	79,832	48x	GCA_020775715.1	Johler et al., 2018	This study
CVUAS2492	Food (lettuce; linked to foodborne outbreak, Germany)	Draft	72,217	89x	GCA_020775795.1	Johler et al., 2018	This study
CVUAS9659	Food (lettuce; linked to foodborne outbreak, Germany)	Draft	67,809	82x	GCA_020775695.1	Johler et al., 2018	This study
CVUAS9660	Food (lettuce; linked to foodborne outbreak, Germany)	Draft	72,795	67x	GCA_020775705.1	Johler et al., 2018	This study
P01_1	Food (honey)	Draft	67,809	79x	GCA_020775875.1	Johler et al., 2018	This study
P01_3	Food (honey)	Draft	67,809	80x	GCA_020775855.1	Johler et al., 2018	This study
CH_684 *	Food (bell pepper)	Draft	96,496	47x	GCA_020775995.1	This study	This study
CH_692 *	Food (cherry tomato)	Draft	65,107	41x	GCA_020775985.1	This study	This study
CH_696	Food (cherry tomato)	Draft	65,107	47x	GCA_020775915.1	This study	This study
CH_698	Food (bell pepper)	Draft	93,257	52x	GCA_020775975.1	This study	This study
CH_709	Food (rocket salad)	Draft	59,222	50x	GCA_020775595.1	This study	This study
CH_722	Food (cherry tomato)	Draft	84,251	41x	GCA_020775865.1	This study	This study
CH_733	Food (cherry tomato)	Draft	70,892	56x	GCA_020775925.1	This study	This study
CH_746 **	Food (rocket salad)	Draft	56,948	48x	GCA_020775565.1	This study	This study
CH_748 **	Food (rocket salad)	Draft	72,163	46x	GCA_020775555.1	This study	This study

* CH_692 and CH_694 originate from the same food sample; ** CH_746 and CH_748 originate from the same food sample.

**FIGURE 1:**
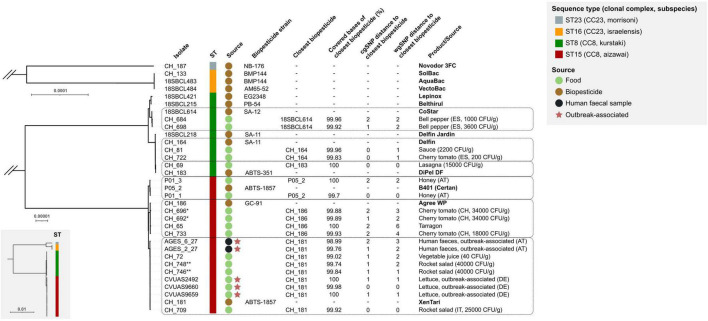
Genetic similarity of *B. thuringiensis* isolates from food, biopesticide products, and outbreaks. Maximum-likelihood phylogenetic trees for CC8 (ST8 and ST15) and CC23 (ST16 and ST23) were constructed from two separate core genome SNP (cgSNP) matrices. Branch lengths between CC8 and CC23 are shown in the full tree (bottom left box). Scale bars indicate the number of variant sites in core genomes of 3.8 Mbp (full tree), 4.6 Mbp (CC8), and 4.1 Mbp (CC23) lengths. The isolates’ sequence type (ST), clonal complex (CC), subspecies, and source of each isolate are labeled according to the legend. Dashed boxes group food/fecal isolates with their putative biopesticide source strain. Whole-genome SNPs (wgSNPs) were identified by mapping sequencing reads from each food/clinical isolate to the genome of the closest biopesticide isolate. The genome coverage refers to the number of bases in the reference biopesticide genome covered by reads of the food/clinical isolate, excluding repeat regions. The phylogeny was visualized in iTOL (Letunic and Bork, 2019). CFU: colony-forming units; AT, Austria; DE, Germany; ES, Spain; IT, Italy. * CH_692 and CH_694 originate from the same food sample ** CH_746 and CH_748 originate from the same food sample.

All 33 genomes contained the *B. thuringiensis*-defining *cry* genes and were assigned to *panC* group IV. The 5 outbreak-associated isolates and 15 food isolates belonged to one of two closely related lineages ST8 (subspecies *kurstaki*) or ST15 (subspecies *aizawai*), which together form CC8. Most (11 out of 15) biopesticide isolates investigated in our study also fell into these two lineages. SNP analyses suggested that all food/fecal isolates originated from one of six distinct biopesticide strains: 19 of the 20 isolates differed by ≤ 4 wgSNPs (≤ 2 cgSNPs) from their phylogenetically closest biopesticide isolate (Figure 1) and food isolate CH_65 differed by 6 wgSNPs (2 cgSNPs) from its closest biopesticide isolate. The food isolates matched with biopesticide strains ABTS-351, ABTS-1857, ABTS-1857/B401, GC-91, SA-11, or SA-12. The five outbreak-associated isolates clustered in a sublineage of ST15 and differed by 0–3 wgSNPs (0–2 cgSNPs) from the biopesticide strain ABTS-1857, suggesting this biopesticide as a highly likely origin. Whereas most biopesticides are applied on a wide spectrum of crops, the biopesticide B401 is specifically recommended for the control of wax moths on honeycombs. Accordingly, the two isolates recovered from honey products corresponded to the B401 isolate (0 and 2 wgSNP/cgSNP difference, respectively).

### Toxin Gene Profiles of Biopesticide Isolates

The toxin gene profile varied between biopesticide isolates (Table 3). All biopesticide isolates except CH_187 (NB-176) carried intact genes and associated 5’ intergenic regions encoding cytotoxin CytK2 (*cytK2*), hemolysin BL (*hblA*, *hblB*, *hblC*, *hblD*), and non-hemolytic enterotoxin (*nheA*, *nheB*, *nheC*). In CH_187 (NB-176), *cytK* was absent and the *nheA* promoter disrupted by a transposase inserted 13 bp upstream of the translation initiation site, likely preventing effective transcription. As expected for *B. thuringiensis*, none of the isolates possessed the *ces* locus (encoding the emetic toxin cereulide).

**TABLE 3:** Presence of intact toxin genes and their 5′ intergenic regions in genomes from biopesticide isolates.

Isolate	Biopesticide strain	ST (CC)	Toxin gene profile			
			*cytK*	*hbl*	*nhe*	*sph*
CH_187	NB-176	23 (CC23)	**–**	+	(+)^a^	+
CH_133	BMP144	16 (CC23)	+	+	+	+
18SBCL483	BMP144	16 (CC23)	+	+	+	+
18SBCL484	AM65-52	16 (CC23)	+	+	+	+
18SBCL421	EG2348	8 (CC8)	+	+	+	**–** ^b^
18SBCL216	PB-54	8 (CC8)	+	+	+	**–** ^b^
18SBCL614	SA-12	8 (CC8)	+	+	+	**–** ^b^
18SBCL218	SA-11	8 (CC8)	+	+	+	**–** ^b^
CH_164	SA-11	8 (CC8)	+	+	+	**–** ^b^
CH_183	ABTS-351	8 (CC8)	+	+	+	**–** ^b^
P05_2	ABTS-1857 (Bt401/402)	15 (CC8)	+	+	+	+
CH_186	GC-91	15 (CC8)	+	+	**+**	**–** ^b^
CH_181	ABTS-1857	15 (CC8)	+	+	+	+

*^a^Promoter region disrupted by a transposase insertion.*

*^b^3′-terminus disrupted by a prophage insertion.*

**FIGURE 2:**
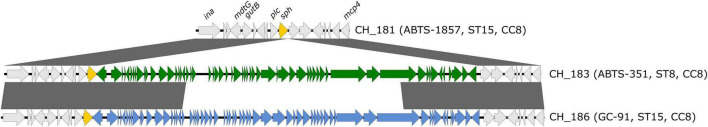
Disruption of the sphingomyelinase-encoding gene *sph* by prophage insertions. Genetic context of *sph* in *B. thuringiensis* biopesticide isolates CH_181 (ABTS-1857, ST15), CH_183 (ABTS-351, ST8), and CH_186 (GC-91, ST15). Shaded boxes between sequences indicate homologous regions (>90% sequence identity). CH_183 and CH_186 harbor the prophages BtCS33 (42 kb, green) and BceA1 (42 kb, blue), respectively, inserted into the 3′-terminus of *sph*. The figure was generated with Easyfig 2.1 (Sullivan et al., 2011).

In multiple *B. thuringiensis* genomes, the *sph* gene (sphingomyelinase) was disrupted by the insertion of prophages (Figure 2 and Table 3). Hybrid assemblies from biopesticide isolates CH_183 (ABTS-351, ST8) and CH_164 (SA-11, ST8) revealed the presence of the intact 42 kb *Bacillus* prophage BtCS33 (NC_018085.1, >99% alignment coverage and identity) inserted into the 3′-terminus of *sph*. Long-read assemblies from isolate CH_186 (GC-91, ST15) generated using Canu or Raven contained the intact 42 kb *Bacillus* prophage BceA1 (GCF_003047795.1, > 99% alignment coverage and identity) inserted into the 3′-terminus of *sph*. By contrast, *sph* was located at the end of a split contig in a hybrid assembly of CH_186 generated with Unicycler. Verification by PCR suggested that both intact and phage-disrupted *sph* loci are detectable in CH_186, possibly resulting from subpopulations of lysogenic, pseudo-lysogenic, lytic, and/or naïve cells. In sequencing read mapping analyses, the average base coverage in prophage regions of CH_186 was > 4-fold higher than in flanking regions, further indicating the presence of extrachromosomal DNA of active bacteriophages. Almost half of the BtCS33 and BceA1 genomes are homologous, including the identical site-specific integrases GP25 and P30, respectively (Figure 2). A 19 bp identical region in *sph* and downstream of the integrase genes was identified as the insertion site. In short-read assemblies, phage insertions could be identified by an incomplete *sph* sequence (96.85% alignment coverage). Accordingly, disrupted *sph* was also detected in all other biopesticide isolates from ST8, whereas biopesticide isolates from CC23 and two out of three biopesticide isolates from ST15 harbored intact *sph* genes. The toxin gene profile (*sph* completeness and *nhe*, *hbl*, *cytK*, *ces*) of food and fecal isolates always corresponded to their respective closest biopesticide isolate (shown in Figure 1).

## Discussion

*B. thuringiensis* is frequently found on fresh vegetables, particularly on tomatoes, and has also been detected in processed foods such as lasagna, ratatouille, or strawberry cake (Johler et al., 2018; Bonis et al., 2021). Reliable source attribution is essential to assess a potential consumer risk posed by *B. thuringiensis*-based biopesticides. In this study, we investigated a potential link between 13 biopesticide isolates and 20 *B. thuringiensis* isolates from food and human fecal samples. SNP analyses revealed that all food/fecal isolates genomically matched (≤ 6 wgSNPs, median 2 wgSNPs; ≤ 2 cgSNPs, median 1 cgSNPs) with one of six widely used biopesticide strains, strongly suggesting biopesticide products as their source. While there is no specific SNP cutoff to define closely related isolates, pairwise distances among outbreak-linked emetic *B. cereus* isolates, for comparison, ranged from 0 to 8 cgSNPs when applying the CFSAN pipeline (Carroll et al., 2019). In some cases, the food/fecal isolates investigated here shared the same wgSNPs: for instance, food isolates CH_65, CH_692, CH_696, and CH_733 had two wgSNPs (CH_186 C3822937T and pCH_186-a T177153C; confirmed by Sanger sequencing) in common that distinguished them from their closest biopesticide isolate CH_186 (GC-91), suggesting that these isolates originate from a GC-91-like *aizawai* biopesticide product not included in this study. Alternatively, the investigated biopesticide isolate CH_186 may have acquired SNPs at these positions, for instance during storage and subculturing in the sequencing laboratory. A single SNP difference was found between the same-sample isolates CH_692 and CH_696, demonstrating that the identification of a limited number of SNPs among same-source isolates is plausible.

Consistent with our finding, the biopesticide residues were detected on food samples that corresponded to the recommended field of application of the respective products: B401, authorized for moth control in beekeeping, was re-isolated from honey; biopesticide strains authorized for pest control on various crops were re-isolated from fruits, vegetables, spices, and processed food; *B. thuringiensis* strains used for mosquito control (*B. thuringiensis* ssp. *israelensis*) or on potato foliage (*B. thuringiensis* ssp. *morrisoni* var. *tenebrionis*) were not found. *B. thuringiensis* isolates from lettuce linked to a foodborne outbreak in 2012 in Germany (EFSA BIOHAZ Panel, 2016) and two *B. thuringiensis* isolates from feces associated with a foodborne outbreak in Austria (Schmid et al., 2016) genomically matched ABTS-1857 (≤ 3 wgSNP; ≤ 2 cgSNP).

Our findings are consistent with other recent genomic analyses of *B. thuringiensis* food isolates. Among 83 *B. thuringiensis* isolates from tomatoes and bell pepper from retailers in Germany, 82 (99%) belonged to the ST8 or ST15 lineages (Frentzel et al., 2020), which also comprise the biopesticide strains commonly used for agricultural pest management. Of 42 isolates that were further characterized by WGS, 25 matched (4 cgSNPs) with one of the two included biopesticide strains, with the remaining 17 isolates possibly originating from biopesticide strains that were not covered by this study (Frentzel et al., 2020). In a comprehensive analysis of outbreak-associated B. thuringiensis isolates from food, Bonis et al. (2021) reported that most isolates phylogenetically clustered (0 – 10 cgSNPs) with biopesticide strains (Bonis et al., 2021). A plausible link between food and biopesticide strains was also established using FT-IR spectroscopy or low-resolution genotyping in earlier studies (Frederiksen et al., 2006; Hendriksen and Hansen, 2006; Zhou et al., 2008; Johler et al., 2018).

*B. thuringiensis* cytotoxicity can vary widely. While many strains exhibit low or medium cytotoxicity, a wild-type strain has been demonstrated to surpass the cytotoxicity of a *B. cereus* outbreak strain by a factor of 1.5 (Johler et al., 2018). Genes encoding the heat-labile enterotoxins Nhe, Hbl, and CtyK were identified in all biopesticide strains except NB-176 (ST23), which lacks the *cytK* gene. In addition, long-read sequencing revealed that the *nhe* promoter region of NB-176 is disrupted by a transposase insertion, likely preventing Nhe expression. In a previous study, NB-176 showed notably lower cytotoxicity in a Vero cell assay, indicating lower enterotoxin levels compared to other tested biopesticide strains (Johler et al., 2018).

Many *B. cereus s.l.* strains produce sphingomyelinase, a virulence factor contributing to *B. cereus* cytotoxicity that was shown to interact synergistically with Nhe and Hbl (Beecher and Wong, 2000; Doll et al., 2013). In biopesticide strains from ST8 and one (out of three) biopesticide strains from ST15, the sphingomyelinase-encoding *sph* gene was here found to be disrupted by bacteriophage insertions. In agreement with this disruption, no sphingomyelinase activity was detected in selected biopesticide isolates from ST8 [CH_164 (SA-11) and CH_183 (ABTS-351)] and in CH_186 (GC-91, ST15), whereas ST15 biopesticide isolates with an intact *sph* gene [CH_181 (ABTS-1857), P05_2 (Bt401)] showed low but detectable sphingomyelinase activities in a previous study (Johler et al., 2018). The presence of prophages integrated into *sph* may result from stochastic effects rather than being a strain-specific characteristic.

This study has notable limitations. No information was available on the prior use of *B. thuringiensis*-based biopesticides on the food samples, and there is no generally accepted SNP cut-off value to define a common origin of *B. cereus* isolates. Likewise, complete epidemiological investigations are needed for outbreak-associated isolates to substantiate our findings. The prevalence of *B. thuringiensis* was assessed on a limited number of samples from few food categories. Larger screenings across a wide range of food categories from various geographical origins are required to determine how frequently consumers are exposed to *B. thuringiensis*. Moreover, in our study, *B. thuringiensis* isolates were identified by phase-contrast microscopy. While this method is recognized as a tool for the identification of *B. thuringiensis* (EFSA BIOHAZ Panel, 2016), it cannot be excluded that additional isolates carried *cry* or *cyt* genes.

In conclusion, this study suggests that *B. thuringiensis* isolated from foods typically stem from biopesticide residues and we were able to genomically match outbreak-associated strains to *Bacillus thuringiensis* biopesticides. In addition, we present high-quality genome assemblies of seven widely used biopesticide strains, which provided novel insights into genetic mechanisms underlying differences in their cytotoxicity phenotype and will facilitate improved source-tracking in future studies.

## Data Availability Statement

Sequencing data and genome assemblies generated as part of this study are available under BioProject PRJNA757250 (https://www.ncbi.nlm.nih.gov/bioproject/?term=PRJNA757250). Genome accession numbers of all investigated isolates are listed in Supplementary Table 2.

## Author Contributions

MB, ME-S, RS, and SJ designed the study. DE, PB, SC, and RS isolated strains and conducted laboratory experiments aimed at further characterization of the strains. MB performed bioinformatic analyses. MB and SJ drafted the manuscript that was revised and approved by all authors.

## Conflict of Interest

The authors declare that the research was conducted in the absence of any commercial or financial relationships that could be construed as a potential conflict of interest.

## Publisher’s Note

All claims expressed in this article are solely those of the authors and do not necessarily represent those of their affiliated organizations, or those of the publisher, the editors and the reviewers. Any product that may be evaluated in this article, or claim that may be made by its manufacturer, is not guaranteed or endorsed by the publisher.

## Footnotes

1
https://github.com/FelixKrueger/TrimGalore

2
www.bioinformatics.babraham.ac.uk/projects/fastqc/

3
https://github.com/tseemann/shovill

4
https://github.com/rrwick/Porechop/

5
https://github.com/tseemann/abricate

6
https://github.com/tseemann/phastaf

7
https://github.com/tseemann/snp-dists

8
sourceforge.net/projects/bbmap/

## Supplementary Material

Supplementary Table 1 Food samples screened for contamination with *B. cereus sensu lato*.

Supplementary Table 2 *B. thuringiensis* isolates included in the genomic analyses.
